# Achilles Tendon Rupture: Can the Tendon Gap on Ultrasound Scan Predict the Outcome of Functional Rehabilitation Program?

**DOI:** 10.7759/cureus.10298

**Published:** 2020-09-07

**Authors:** Islam Mubark, Amr Abouelela, Swati Arya, Donald Buchanan, Mosab Elgalli, Jennifer Parker, Neil Ashwood, Charalampos Karagkevrekis

**Affiliations:** 1 Trauma and Orthopaedics, University Hospitals Derby and Burton, Derby, GBR; 2 Trauma and Orthopaedics, University Hospitals of Derby and Burton NHS Foundation Trust, Burton, GBR; 3 Trauma and Orthopaedics, University Hospital of Derby and Burton NHS Foundation Trust, Burton, GBR

**Keywords:** achilles tendon, rupture, ultrasound, gap, rehabilitation

## Abstract

Background and objectives

There is a growing use of functional rehabilitation programs for the treatment of Achilles tendon rupture. Factors such as patient age and level of activity have been used to guide the decision. One of the debated indications is the gap size between the ruptured ends of the tendon. This study aims to define any correlation between the amount of the initial gap between tendon ends and patients outcome treated with the functional rehabilitation program.

Method

A prospective case series study of all patients with acute Achilles tendon rupture treated non-surgically with the functional rehabilitation program between 2016 and 2018. The tendon gap was measured with an ultrasound scan on the initial presentation. Patients were followed for a minimum of 12 months and assessed for Achilles Tendon Rupture Score (ATRS), plantarflexion strength, and re-rupture rate.

Results

A total of 56 patients completed one-year follow-up, and 2 patients had re-ruptures. The mean plantar flexion gap was 13.7 mm. The mean ATRS at 12 months was 85.12. There was no statistically significant correlation between the final ATRS and the mean rupture gap.

Conclusion

The outcome following non-operative functional rehabilitation treatment of rupture Achilles tendon did not correlate with the size of the tendon gap, and authors recommend that decision on functional rehabilitation should not be based on these criteria.

## Introduction

Despite the increased incidence of Achilles tendon rupture over recent years, the optimal treatment for this injury remains controversial [1]. The aim of treating Achilles tendon ruptures is to achieve a rapid return to the pre-injury level of activity with the least complications. 

The treatment can be grouped into surgical and non-surgical methods. Despite a lower re-rupture rate of open surgical repair, it has always been associated with a higher than usual wound-related complication [2]. This has veered many surgeons towards minimal percutaneous repairs which had the benefit of a relatively low re-rupture rate, while also reducing the rates of soft-tissue complications [3]. The non-operative treatment of rupture tendon Achilles has historically been associated with higher rates of re-rupture, ankle stiffness, and tendon weakness. These complications were usually related to the use of plaster immobilization for prolonged periods.

The introduction of new functional rehabilitation programs in the form of protected early weight-bearing and early controlled movement in an orthosis has produced about equal satisfactory results to surgical treatment [4]. There is considerable variation among such protocols in terms of indications such as patient age and level of activity. There have been varying reports regarding the amount of accepted gap between ruptured tendon ends that would benefit from functional rehabilitation [5,6].

In this study, we have been trying to find any correlation between the tendon rupture gap and the patients' outcome following treatment with the functional rehabilitation program and whether the size of this gap should be relied on to assign patient to surgical versus non-surgical treatment.

## Materials and methods

This is a prospective study of all patients with acute Achilles tendon ruptures treated using the functional rehabilitation program between January 2016 and February 2018 in Queens Hospital Burton and completing a minimum of 12-month follow-up.

The patients included were all adults aged over 18 years, sustaining a closed Achilles tendon rupture and presenting within two weeks after the injury.

**Figure 1:**
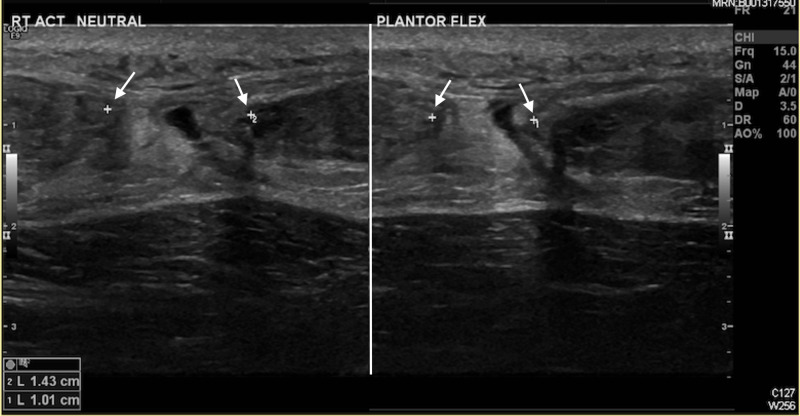
Ultrasound scan of a ruptured Achilles tendon measuring gap between ruptured ends (arrows) in both neutral position and plantar flexion.

In the emergency department, all patients presenting with suspected Achilles tendon rupture were treated initially in a walker boot and referred for an ultrasound scan. The tendon rupture gap was measured in ankle plantar flexion and neutral position (Figure 1).

All the patients, meeting these criteria, were assigned for the functional rehabilitation program. The functional rehabilitation program entailed immediate weight-bearing mobilization in boots (OSSR Rebound® Air Walker Boot, Ossur (UK) Ltd, Stockport, England) with build-up three wedges starting at 30 degrees of plantar flexion. 

Patients were reviewed by physiotherapist every two weeks for removal of single wedge until completing 12 weeks and achieving 0-degree plantar flexion. After boot removal, patients were started on the Achilles tendon strengthening program. Patients were followed up at 3, 6, and 12 months for assessment of Achilles Tendon Rupture Score (ATRS) [7].

A total of 56 patients who satisfied the inclusion criteria and completed 12-month follow-up were included in the study, while those with delayed presentation over two weeks presenting with open injuries or re-rupture were excluded.

## Results

Statistical analysis was performed using Statistical Package for the Social Sciences (SPSS) version 23 (IBM Corp., Armonk, NY). The Pearson correlation coefficient analysis was used to correlate the ATRS and plantar flexion strength with the plantarflexion tendon gap. 

A total of 56 (21 female and 35 males) patients completed 12-month follow-up with a mean follow-up of 15.6 months (range 12-19). The mean age was 56.5 years (range 27-81). The mean plantar flexion gap was 13.7 mm (range 0.67-38.0, SD ± 7.7). 

**Figure 2:**
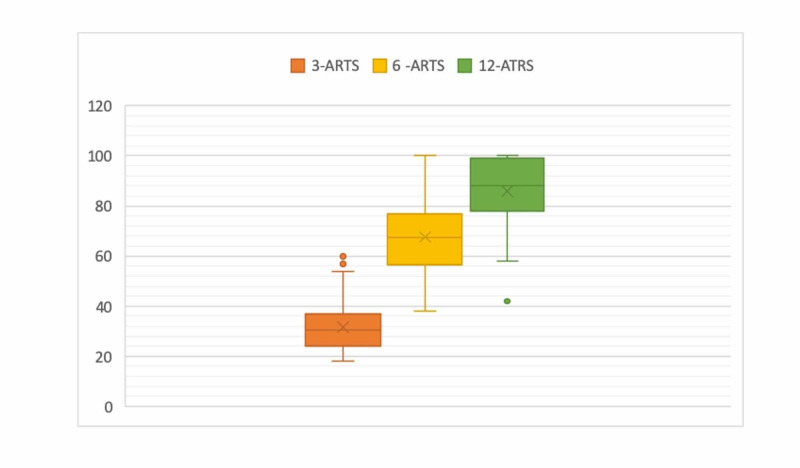
Mean ATRS (Achilles Tendon Rupture Score) at 3, 6, and 12 months.

There were three re-ruptures (3.5%), all occurred after 12-month post-injury. At 12 months, the mean ATRS was 85.12 (range 32-100, SD ± 12) (Figure 2).

**Figure 3:**
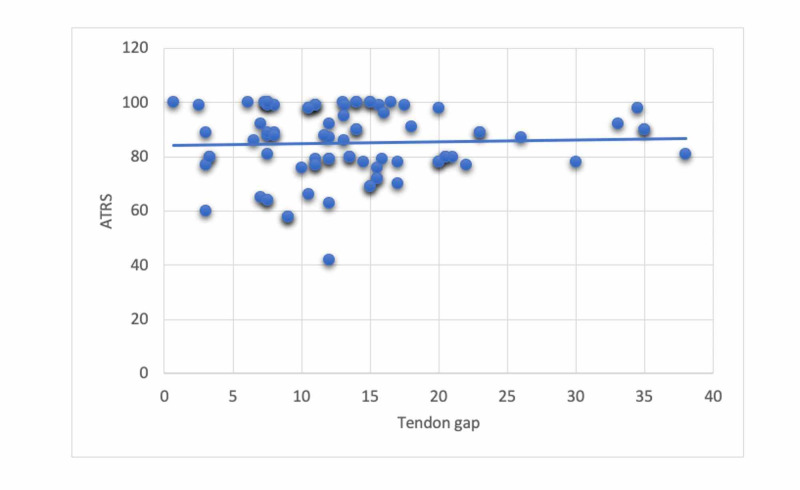
Regression analysis between tendon gap and ATRS (Achilles Tendon rupture Score) at 12 months showing no significant correlation between the two variables.

There was no statistically significant correlation between the ATRS and average tendon gap measurement (Pearson correlation = 0.091, P = 0.167) (Figure 3).

## Discussion

Rupture of the Achilles tendon is one of the most common tendon injuries in the adult population. The incidence of this injury is increasing as more aging population engages in recreational activities [8]. The decision on the management of ruptured Achilles tendon has always been balanced between the risks of wound problems with surgery and a re-rupture risk with non-surgical treatment [9]. The non-surgical treatment of Achilles tendon rupture historically involved a long period of cast immobilization. Biomechanical studies have shown that early mobilization improves the quality of tendon healing and decreases its susceptibility to re-rupture [10,11]. These findings encouraged the introduction of the concept of early mobilization and functional rehabilitation programs in the management of Achilles tendon rupture. Willits and colleagues in their landmark paper in 2010 showed no significant difference in the re-rupture rate between operative and non-operative treatment using functional rehabilitation program [12]. 

The size of the tendon gap on an ultrasound scan has been used as a tool for the decision regarding the suitability of functional rehabilitation with variable reports on the accepted gap and related outcome. It seems that the cut limit of the size of tendon gaps considered in these studies is arbitrary and anecdotally proposed. The obliteration of tendon gap on plantar flexion has been selected as encouraging criteria for successful functional rehabilitation and the presence of a gap of more than 5 or 10 mm has been used to support operative treatment [13]. In one study on 45 patients, Westin et al. reported a higher re-rupture rate in non-surgically treated patients when the gap on ultrasound scan was more than 10 mm and there were significantly worse outcomes in patients with a diastasis of >5 mm. All patients in their series were not allowed weight-bearing for the first six weeks [6]. Lawrence et al., reporting on 38 patients treated non-operatively, showed that patients with a gap <10 mm had significantly greater strength than those with gaps >10 mm. However, there was no difference in ATRS between the two groups [14]. Kotnis et al. in a comparative study allocated patients with a gap of 5 mm or more in equines on ultrasound to have surgery and those with a gap of less than 5 mm to receive non-operative treatment. There was no statistically significant difference between the two treatment groups regarding the incidence of complications, but the study did not compare any validated patient outcome measures [15].

In our study, we tried to define the direct correlation between gap size and the patient outcome and whether there is a strong correlation to justify using the tendon gap size on ultrasound scan for recommending the functional rehabilitation program. This study has limitations regarding the small number of patients in the study and the short duration of follow-up (12 months), which did not allow us to address the long-term complications.

## Conclusions

The study did not show a statistically significant correlation between the tendon gap size and ATRS at 12 months. These results indicate that the size of the tendon gap on the ultrasound scan should not be relied upon as selection criteria for a functional rehabilitation program.
